# Impact of Free Sugar Consumption on Dental Caries: A Cross-Sectional Analysis of Children in the United States

**DOI:** 10.3390/dj13020048

**Published:** 2025-01-22

**Authors:** Val Joseph Cheever, Amir Mohajeri, Kavina Patel, Richard Collin Burris, Man Hung

**Affiliations:** 1 College of Dental Medicine, Roseman University of Health Sciences, South Jordan, UT 84095, USA; 2 Division of Public Health, University of Utah, Salt Lake City, UT 84108, USA

**Keywords:** free sugar, dental caries, children, socioeconomic

## Abstract

**Background/Objectives:** The excessive consumption of free sugars adversely impacts health, contributing to systemic disorders and significantly increasing the risk of dental caries. Children are particularly vulnerable to dental caries due to their dietary habits and oral hygiene practices. This study aimed to examine the relationship between sugar intake and dental caries experience in a sample of children aged 6–12 years. **Methods:** Data were analyzed from the National Health and Nutrition Examination Survey (NHANES) covering the years 2011–2016. This cross-sectional study utilized nationally representative data from NHANES, which provides comprehensive health assessments on the U.S. population. This study focused on 3658 children aged 6–12 years. Free sugar intake and its association with dental caries were evaluated using the Decayed, Missing, and Filled Teeth (DMFT) index, a widely accepted measure for assessing cumulative caries experience in permanent teeth. Demographic and socioeconomic factors were also accounted for. The sample primarily consisted of generally healthy children, with mild to moderate health conditions included in the analysis. **Results:** The average Decayed, Missing, and Filled Teeth (DMFT) score was 1.69 (SD = 2.61). Untreated dental caries affected 19.1% of the sample, with disproportionately higher rates observed among Mexican American children (23.8%), children from lower socioeconomic backgrounds (24.2%), those with less-educated parents (25.1%), and overweight children (22.9%). Mean daily free sugar intake was 72.46 g (SD = 50.45), with significant variations across race/ethnicity, parental education, and gender. A statistically significant association was found between free sugar intake and dental caries in U.S. children (*p* < 0.001). **Conclusions:** Free sugar intake is strongly associated with an increased risk of dental caries. Disparities in caries prevalence were evident based on race/ethnicity, socioeconomic status, and parental education levels. Regulating sugar intake and promoting dietary education are effective strategies to reduce the risk of dental caries and improve oral health outcomes among children.

## 1. Introduction

The average American lifestyle is characterized by a predominantly sedentary routine and a diet that is often less than optimal. This diet typically includes an excessive intake of calories, fats, and free sugars, contributing to high rates of health issues such as obesity and diabetes. Free sugars, as defined by the World Health Organization (WHO), are sugars added to foods or naturally present in syrups, honey, and concentrated fruit juices, but that exclude lactose found in milk and milk products [1]. These sugars should be restricted for health reasons, as they are a significant dietary concern [2,3]. The WHO recommends that adults and children consume no more than 30 g and 19 g of free sugars per day, respectively [1]. In 2024, the American Association of Pediatric Dentistry (AAPD) recommended reducing sugar consumption to less than 10% of total energy intake for children aged 4–8, 16 g of sugar or less [4]. Despite these recommendations, studies have shown that the average daily free sugar intake is far higher than these guidelines, with a mean intake of 193 g/day in some studies [5]. This large discrepancy is concerning, particularly considering the strong associations between sugar consumption and obesity, diabetes, and dental caries.

Dental caries remains one of the most common chronic conditions affecting children in the U.S. [6]. According to the Centers for Disease Control and Prevention (CDC), approximately 3% of children aged 6–11 years have at least one permanent tooth with untreated decay. Among children with one or more decayed, missing, or filled permanent teeth (DMFT > 0), the average includes 0.3 decayed teeth and 1.6 filled teeth [7]. The mean number of filled teeth varied slightly across different sociodemographic groups, ranging from 1.4 to 1.9 teeth [7]. During 2015–2016, 45.8% of children aged 2–19 experienced caries, and 13.0% had untreated cavities. Caries prevalence increased with age, rising from 21.4% in children aged 2–5 to 53.8% in those aged 12–19 [8]. A recent systematic review underscoring the prevalence of dental caries in children highlights its widespread impact, offering critical insights for public health initiatives, dental practice recommendations, and research priorities aimed at preventing and managing this significant oral health challenge in younger populations [9]. The excessive consumption of free sugars has been closely linked to weight gain. According to the National Center for Health Statistics, about two-thirds of the United States (U.S.) adults and one-third of children aged 2 through 19 years are classified as overweight or obese [2]. In addition to obesity, diabetes is a major concern, with an estimated 23.6 million Americans affected. Alarmingly, about 5.7 million of these cases remain undiagnosed, indicating gaps in awareness and access to healthcare [10]. The rising rates of obesity and diabetes significantly contribute to the prevalence of chronic diseases and premature deaths, elevating them to the level of a public health crisis [11]. These conditions not only impose a substantial burden on individuals and families but also strain healthcare systems, highlighting the urgent need for preventive measures and targeted interventions to address these growing challenges. However, while the systemic health effects of free sugar consumption have been widely studied, its specific role in promoting disparities in oral health outcomes, particularly among vulnerable pediatric populations, remains underexplored.

In addition to contributing to systemic diseases, free sugar is a major contributor to oral health issues. High sugar consumption promotes an acidic oral environment that increases susceptibility to dental caries [12]. Sugars and carbohydrates are hydrolyzed by salivary amylase, providing oral bacteria with a substrate to metabolize, which in turn lowers the salivary pH [13]. This acidic environment facilitates the demineralization of enamel and dentin by causing the loss of calcium and phosphate ions from hydroxyapatite crystals [14]. If left untreated, this process leads to the development and progression of dental caries. Free sugars, particularly sucrose, are closely linked to the initiation and advancement of dental caries lesions [15,16]. Sucrose is naturally present in fruits and vegetables, but it is also commonly added to processed foods and beverages such as sodas, cakes, and milkshakes. While adults frequently consume products high in free sugars, foods and drinks marketed toward children often contain substantial amounts of free sugar, further exacerbating the risk of oral health problems in this vulnerable population.

Children are particularly susceptible to developing dental caries due to their evolving oral hygiene practices and dietary habits [17]. Key factors contributing to the high prevalence of childhood caries include socioeconomic background, parental education levels, and limited access to dental care [17,18]. Families from lower socioeconomic backgrounds often face barriers to accessing preventive dental services and may lack the resources or knowledge needed to establish healthy oral hygiene routines [19]. Free sugars, commonly found in sweetened beverages, cereals, and candies, play a significant role in the development of caries [18]. Early exposure to free sugar not only contributes directly to tooth decay but also influences children’s taste preferences, leading them to favor sugary foods and beverages over healthier alternatives [15]. This dietary pattern increases the risk of caries and can establish habits that persist into adulthood. Without proper education on diet and oral hygiene from parents or dental professionals, children are less likely to develop effective oral care habits [20]. Poor oral hygiene in childhood not only impacts the health of primary teeth but also jeopardizes the health and development of permanent teeth, potentially leading to long-term oral health issues [21]. Promoting early intervention and parental education is essential to mitigating these risks and fostering lifelong oral health [22,23]. Despite the known link between dietary habits and oral health, there remains a lack of comprehensive research that investigates the interaction between sugar consumption, socioeconomic factors, and disparities in dental caries prevalence among children. Addressing these gaps is critical for developing effective, equity-focused public health strategies.

Sucrose is widely believed to be the most cariogenic sugar. Sucrose enhances plaque accumulation by supporting the synthesis of extracellular glucans through *Streptococcus mutans* [24]. Glucan alters plaque porosity, allowing deeper penetration of sugars and acid production on the tooth surface and thus causing dental caries [24]. Sucrose is also naturally found in milk, fruits, vegetables, and grains. These natural sugars do not make an important contribution to the development of caries due to their components such as calcium, fiber, and water content [16,25]. These components impact salivary flow to help reduce the potential risk of the sugars. The World Health Organization classifies sugars other than natural sugars as free sugars that should be restricted for health [16,26]. Restriction of free sugars, especially at a young age, can influence future U.S. rates of systemic health and dental caries. Although efforts have been made to understand the cariogenic properties of sucrose and other free sugars, there is limited research addressing the effectiveness of existing dietary recommendations in mitigating the prevalence of dental caries, particularly within socioeconomically disadvantaged populations.

The connection between free sugar consumption and the heightened risk of dental caries underscores the importance of controlling sugar intake to prevent the progression of oral health diseases [12,24]. This study specifically aimed to explore the effects of sugar consumption on the prevalence of dental caries in a sample group of children aged 6 to 12 years. The primary hypothesis of the study was that increased free sugar consumption would result in a higher prevalence of dental caries among children in the U.S. The study also aimed to investigate key confounding factors, including socioeconomic background, race, ethnicity, and access to education, to provide a broader understanding of how these factors might influence the relationship between sugar intake and caries risk. These factors provide a holistic perspective on the risk profile of U.S. children and offer insights into disparities that might affect oral health outcomes.

Dental caries remains one of the most prevalent chronic diseases in children, significantly impacting their overall health, quality of life, and academic performance. Despite advancements in dental care, disparities in oral health outcomes persist, particularly among underserved and socioeconomically disadvantaged populations. This research sought to fill a critical gap by identifying the role of free sugar and its interaction with various socioeconomic factors in influencing childhood dental caries. By providing insights into these disparities, the study aimed to contribute to the development of targeted interventions and policies to reduce the burden of dental caries in U.S. children.

## 2. Methods

This study followed the Strengthening the Reporting of Observational Studies in Epidemiology (STROBE) checklist [27] for cross-sectional studies. The methods were outlined to ensure transparency and thorough reporting, in line with the recommendations of the STROBE guidelines (Supplementary Table S1).

### 2.1. Study Design

This study used cross-sectional data from three cycles of the National Health and Nutrition Examination Survey (NHANES) (2011–2016) to enhance subgroup estimates. The NHANES collects data from a nationally representative U.S. sample using multi-stage probability sampling, with interviews, physical assessments, and laboratory examinations to provide comprehensive health and nutrition data.

The study received ethical approval from the Ethics Review Board of the National Center for Health Statistics Research under Protocol #2011–17 for the 2011–2012 cycle, with subsequent continuation for the 2013–2014 and 2015–2016 cycles. Written parental consent was obtained for participants under 18 years of age. In each NHANES cycle, approximately 10,000 individuals participated, undergoing home interviews and health evaluations at mobile examination centers. Specifically, there were 9756 participants in 2011–2012 (response rate: 72.6%), 10,175 in 2013–2014 (response rate: 71.0%), and 9971 in 2015–2016 (response rate: 61.3%). Detailed information about NHANES methodologies and protocols is available on the Centers for Disease Control and Prevention website (https://wwwn.cdc.gov/nchs/nhanes/Default.aspx).

### 2.2. Setting

Data for this study were collected from mobile examination centers (MECs), which are temporary, mobile facilities equipped for conducting health assessments and examinations. These centers were set up at various locations across the U.S. during the NHANES cycles, providing a convenient way to collect data from participants nationwide. Recruitment occurred during home interviews, followed by health evaluations at the MECs. The assessment included interviews, physical assessments, and laboratory examinations. The study covered the periods of data collection from 2011 to 2016.

### 2.3. Participants

The study focused specifically on children between the ages of 6 and 12 who had complete information regarding both free sugar consumption and oral health examination, resulting in a sample of 3658 participants. Inclusion criteria were children within the specified age range and for which there was available information on both dietary habits and dental health. Exclusion criteria were applied to participants who did not have complete data on sugar intake or oral health assessments. The participants were generally healthy, with mild to moderate medical conditions controlled for in the analysis, based on their BMI and other health-related measures. No exclusions were made based on the presence of medical conditions. This age range was selected due to its relevance to key developmental, behavioral, and social factors that could impact oral health outcomes [28]. This period includes the mixed dentition stage, which is marked by the transition from primary to permanent teeth, and is associated with increased susceptibility to dental caries due to factors such as new tooth eruption and changes in oral hygiene practices. While the mixed dentition stage may contribute to heightened caries activity, this age group provides valuable insight into the relationship between free sugar intake and early dental health.

### 2.4. Variables and Data Sources/Measurement

In this study, dental caries was assessed by certified dentists who had received training in NHANES methodologies. The examinations took place at the MEC using specialized equipment including artificial lighting, a portable dental chair, and compressed air. The study employed the DMFT index as the key outcome measure, which was specifically applied to permanent teeth. The DMFT index was chosen because it is more relevant for individuals beyond the age of 6 when primary dentition transitions to permanent dentition [29,30]. Permanent teeth are more accessible and may provide a more comprehensive understanding of long-term oral health. DMFT was determined based on the ‘Coronal Caries: Tooth Count’ component of the dental examination, wherein it was calculated as the cumulative count of designated codes (E, J, K, M, P, Q, R, T, X, and Z). In the NHANES, data for primary and permanent dentitions are reported separately, with “dmft” used for primary teeth and DMFT for permanent teeth. To ensure accuracy and consistency, inter-examiner reliability was assessed by having a reference examiner re-examine a portion of participants. The agreement between the original and reference examiners’ measurements was quantified using kappa and intraclass correlation coefficients, with blinded evaluations to minimize bias and ensure standardized judgments [31]. The NHANES plan and operations manual offers extensive descriptions of caries scoring criteria, along with quality assurance measures and training/calibration protocols.

At the MEC, a 24 h dietary recall interview was administered to assess the child’s dietary intake, encompassing all food items and beverages. To quantify free sugar consumption for each NHANES cycle, the Food Patterns Equivalent Database (FPED), sourced from the U.S. Department of Agriculture (USDA), was utilized [32]. This database quantifies free sugars in teaspoon equivalents (tsp. eq.), with one teaspoon equivalent defined as 4.2 g of sugar, equivalent to the sugar content in one teaspoon of granulated sugar. According to FPED definitions, free sugars encompass sugars, syrups, or caloric sweeteners incorporated into foods and beverages during processing or home preparation, as well as those added at the table (e.g., sugar added to coffee or tea). The child’s free sugar intake was stratified into quartiles for subsequent analysis.

The covariates incorporated into our analyses comprised age, gender, race/ethnicity, family educational level, income, body mass index (BMI), and access to oral health services. Income was evaluated through the ratio of family income to localized poverty threshold levels, which divides a family’s total household income by the poverty guidelines specific to their household size, state, and year. A poverty-to-income ratio (PIR) of less than 1 indicates that a person or family is in poverty, while a PIR of 1 or greater indicates that they are not [33]. Access to oral health services was evaluated with two specific questions. The first question was, ‘When did you last visit a dentist?’, with response options categorized into ‘Within last year’, ‘More than 1 year but less than 5 years’, and ‘More than 5 years, never, or does not know’. The second question was, ‘During the past 12 months, was there a time when you needed dental care but could not get it at the time?’, with response options ‘Yes’ or ‘No’. BMI was categorized based on sex- and age-specific percentiles according to the sex-specific 2000 BMI-for-age growth charts developed by the CDC for the U.S. child population [34]. The categories of the covariates are presented in Table 1.

### 2.5. Bias

Efforts to address potential sources of bias included controlling for several confounding variables such as age, gender, race/ethnicity, family education level, income, BMI, and access to oral health services. These factors were accounted for to minimize bias in the observed association between free sugar intake and dental caries.

### 2.6. Study Size

A total of 3658 children aged 6–12, who had complete data on both free sugar intake and oral health assessments, were included in the study. This sample size was derived from available NHANES data for the specified age range and the variables of interest.

### 2.7. Quantitative Variables

Quantitative variables, including DMFT scores and free sugar intake, were analyzed by stratifying free sugar intake into quartiles. DMFT scores were compared across sociodemographic groups using non-parametric tests (Mann–Whitney U test and Kruskal–Wallis test, as appropriate).

### 2.8. Statistical Methods

Data analysis was conducted using SPSS version 29. To assess the association between free sugar intake and dental caries, we first evaluated the distribution of the DMFT scores using the Shapiro–Wilk test for normality. Since the DMFT scores were not normally distributed (*p* < 0.05), non-parametric tests were used for subsequent analyses. The Mann–Whitney U test and the Kruskal–Wallis test were employed to compare DMFT scores across different sociodemographic groups.

To evaluate the association between free sugar intake and dental caries experience, free sugar intake was initially treated as a continuous variable. However, to assess potential non-linear associations and better capture variations in the data, we divided the free sugar intake into four quartiles. This allowed us to examine differences in dental caries experience across distinct levels of free sugar intake.

Additionally, to examine the linear relationship between continuous free sugar intake and DMFT scores, we performed Spearman rank correlation due to the non-normal distribution of the DMFT data. This test helped to further assess the strength and direction of the relationship between the two continuous variables.

Poisson regression models were initially employed to examine associations between free sugar intake and DMFT scores, treated as a count variable. However, given evidence of overdispersion, we employed Negative Binomial regression models, which better account for the greater variance observed in the data. The models were adjusted for potential confounders, including age, gender, race/ethnicity, family educational level, income-to-poverty ratio, BMI, and access to oral health services. Results are presented as rate ratios (RR) with 95% confidence intervals.

## 3. Results

### 3.1. Study Population and Descriptive Statistics

We analyzed data from 3658 children aged 6–12 years from three continuous NHANES cycles (2011–2012, 2013–2014, and 2015–2016) to evaluate dental health and its association with socioeconomic and dietary factors. The mean DMFT score among the sample was 1.69 (±SE = 0.04). The mean age was 8.90 years (±SE = 0.03), and boys constituted 50.8% of the sample population.

### 3.2. Bivariable Analysis of DMFT Scores

Table 1 presents the bivariable analysis outcomes for mean DMFT scores. Boys (Mean = 1.80, ±SE = 0.06) had higher average DMFT scores than girls. Significant differences in mean DMFT scores were also observed based on race/ethnicity, parental education, poverty income ratio, and access to oral health services. Mexican American children (Mean = 2.16, ±SE = 0.11), children whose parents had low educational attainment (Mean = 2.26, ±SE = 0.15), and those from families with lower socioeconomic status (Mean = 2.12, ±SE = 0.08) exhibited notably higher DMFT scores. Access to dental care was another significant factor. Children who were unable to obtain dental care in the previous year had the highest DMFT scores (Mean = 2.6, ±SE = 0.2). Similarly, children who had not visited a dentist in over a year but less than five years also had elevated DMFT scores (Mean = 1.77, ±SE = 0.13). In contrast, body mass index (BMI) was not significantly associated with DMFT scores.

### 3.3. Association Between Free Sugar Intake and DMFT Scores

Table 2 demonstrates the association between free sugar intake and DMFT scores. A higher intake of free sugar was positively correlated with increased DMFT scores. Children in the highest quartile of free sugar intake had the highest mean DMFT scores (mean = 1.94, ±SE = 0.09). In crude models, these differences were statistically significant, and the relationship persisted after adjusting for potential confounding factors. The RR of higher DMFT scores was significantly elevated for the second quartile (RR = 1.22; 95% CI 1.08–1.38), third quartile (RR = 1.18; 95% CI 1.04–1.34), and fourth quartile (RR = 1.39; 95% CI 1.23–1.58) of free sugar intake. These findings demonstrate a dose–response relationship, with greater sugar intake being associated with higher DMFT scores.

Furthermore, Spearman rank correlation analysis showed a significant positive correlation between free sugar intake and DMFT scores (ρ = 0.047, *p* < 0.05), further supporting the observed association. As free sugar intake increased, so did the dental caries experience, reinforcing the observed trends in the quartile-based analysis.

## 4. Discussion

This study aimed to explore the association between free sugar intake and dental caries in school-aged children, focusing on sociodemographic factors such as income, ethnicity, and parental education. Our hypothesis was supported, as we found a strong and consistent association between higher free sugar intake and increased dental caries prevalence, as measured by DMFT scores. Children in the highest quartiles of sugar consumption consistently exhibited elevated DMFT scores across three NHANES data cycles (2011–2016). Additionally, significant disparities in caries prevalence were observed, with children from low-income households, Mexican American children, and those with less-educated parents exhibiting higher caries rates.

The findings of this study underscore the critical role of dietary habits, particularly free sugar intake, in the pathogenesis of dental caries. The link between free sugar consumption and caries aligns with findings from previous research [18]. Sugar consumption has long been recognized as a significant risk factor for dental caries due to its interaction with oral bacteria producing acids that demineralize tooth enamel [35]. The strong association observed between sugar intake and elevated DMFT scores highlights the importance of addressing sugar consumption as a key component of caries prevention strategies.

Globally, similar associations have been reported. For example, studies from Brazil [15] and the United Kingdom [36] have demonstrated a direct link between sugar consumption and dental caries in children, emphasizing the universality of this risk factor. A study in India found that higher sugar intake was significantly associated with poor oral health outcomes, particularly in children from socioeconomically disadvantaged backgrounds [37]. These findings highlight that the issue is not confined to the U.S. but reflects a global challenge in oral health management. However, the severity of disparities and access to preventive care vary widely across countries, with many low- and middle-income nations facing additional barriers related to healthcare infrastructure and public health policies [38].

Furthermore, this study reveals that sociodemographic factors contribute significantly to the disparities observed in oral health outcomes. Children from lower socioeconomic backgrounds, those with less-educated parents, and minority populations—particularly Mexican Americans—are disproportionately affected by dental caries. These findings align with the existing literature, which has shown that low-income families often have limited access to healthy food options and are more likely to consume sugar-laden foods and beverages, exacerbating the risk of dental caries [19,39,40,41]. For instance, similar disparities have been reported in studies from South Africa, where children in rural areas exhibit higher caries prevalence due to limited access to affordable, nutritious food and preventive dental care [42].

Parental education level is a key socioeconomic factor that influences the ability to make informed, healthy lifestyle choices [43]. For example, parents with higher levels of education tend to have more positive attitudes and stronger intentions to limit their children’s sugar intake compared to less-educated parents [44]. One study found a significant link between parental education level and dental caries, showing that children of highly educated parents had lower dental caries indices, such as DMFT, compared to those whose parents had lower education levels [45]. This supports our findings that children with less-educated parents are more vulnerable to dental caries. The association between parental education levels and children’s oral health outcomes indicates a need for broader educational initiatives. Incorporating oral health education into school curriculums and community workshops can empower families with the knowledge to make healthier dietary and hygiene choices. Collaborations between schools, healthcare providers, and community organizations could create comprehensive programs that address both education and access issues.

Mexican American children are particularly susceptible to dental caries due to various factors, including socioeconomic status [46], neighborhood conditions [47], acculturation [48], fatalism [48], living arrangements [48], and access to preventive dental care [48]. These disparities highlight the need for targeted public health strategies that address the unique challenges faced by Mexican American communities, such as improving access to affordable dental care, promoting preventive measures, and addressing socioeconomic and cultural barriers to better oral health outcomes.

This study also emphasizes the importance of improving access to oral healthcare services. The findings suggest that children who lack access to regular dental care are at a higher risk of developing untreated dental caries. One key preventive behavior is consistent dental visits, which allow dentists to evaluate a child’s caries risk, offer anticipatory guidance based on risk factors, implement strategies to reduce these risks, and provide preventive treatments such as fluoride applications and sealants [5]. An observational study demonstrated that providing at least four fluoride varnish treatments during medical well-child visits significantly reduced tooth decay rates among American Indian children [49]. Similarly, school-based fluoride programs in New Zealand have been effective in reducing caries rates, showcasing the impact of community-focused preventive care across different cultural and healthcare contexts [50]. Policies that expand access to affordable dental care, such as increasing funding for public health insurance programs like Medicaid and the Children’s Health Insurance Program, could help bridge this gap. School-based oral health initiatives, such as dental sealant programs and fluoride varnish applications, could also play a critical role in reducing the prevalence of dental caries among children, especially those in low-income communities.

It is important to note that the mean daily free sugar intake reported in this study (72.46 g) is far higher than the 2024 recommendations by the AAPD, which advocate for reducing free sugar consumption to less than 10% of the total energy intake, or less than 16 g of sugar for children aged 4–8 [4]. The rise in free sugar intake may be influenced by portion sizes, as larger servings can contribute to higher free sugar consumption [51]. Implementing strategies to manage portion sizes in places like restaurants, cinema shops, and schools could play a vital role in controlling sugar intake. Additionally, nutrition education and policy initiatives should focus on specific foods, such as pizzas and soft drinks, which are frequently consumed by children and adolescents in the United States and contribute to an increased intake of energy, fats, and free sugars [51].

These findings highlight a significant gap between the actual sugar intake and current health guidelines, underscoring the urgent need for public health interventions to reduce excessive sugar consumption and promote healthier dietary habits [52].

These results are important because they not only provide evidence of the direct link between sugar consumption and dental caries but also highlight the need for interventions that address both individual behaviors and broader societal inequities. By identifying vulnerable populations, such as children from low-income families and those with less-educated parents, this study provides a foundation for targeted public health campaigns and clinical practices aimed at reducing caries prevalence. The consistency of these findings with international data highlights the global relevance of addressing sugar consumption and oral health disparities as critical public health priorities.

This study’s most significant contribution to the literature lies in its identification of a dose–response relationship between sugar intake and caries prevalence in U.S. children, supported by nationally representative data across multiple cycles. Moreover, its exploration of sociodemographic moderators, such as ethnicity, income, and parental education, provides a better understanding of how systemic inequities amplify the impact of dietary risk factors. These findings lay the groundwork for targeted public health policies and intervention strategies aimed at reducing disparities and improving oral health equity.

The clinical relevance of these findings lies in informing preventive strategies and clinical practices aimed at reducing dental caries prevalence, especially among vulnerable populations. Dental professionals can integrate dietary counseling into routine check-ups, focusing on reducing free sugar intake and promoting preventive treatments such as fluoride varnish and dental sealants. Additionally, public health policies that improve access to dental care and reduce sugar consumption could further reduce disparities in oral health outcomes.

While this study provides valuable insights into the relationship between free sugar intake and dental caries, several limitations must be acknowledged. First, the use of U.S.-specific data limits the generalizability of the findings to other countries and cultural contexts. Global studies are needed to validate these results in diverse populations. Second, the reliance on 24 h dietary recall interviews introduces the possibility of recall bias, which could affect the accuracy of reported sugar intake. Future studies could address this limitation by utilizing more precise dietary assessment methods, such as food diaries or biomarkers of sugar intake.

Additionally, the use of BMI as a health proxy may oversimplify the relationship between obesity and overall health, as it does not account for variations in body composition. Incorporating alternative measures of obesity, such as dual-energy X-ray absorptiometry or skinfold thickness, could provide a more accurate assessment of body fat and its potential impact on oral health outcomes. Furthermore, no analysis for the primary DMFT was conducted, as children in this age group are still in the mixed dentition stage, and at age 6, some children may not yet have any permanent teeth erupted. Future research should consider both primary and permanent DMFT indices to provide a more comprehensive assessment of dental caries.

This study’s strengths include its large sample size of 3658 children and the use of comprehensive NHANES data, which integrate standardized physical assessments and detailed dietary analyses. The robust design and high-quality data enhance the validity of the findings.

Future research should expand the scope to include populations from diverse geographic and cultural backgrounds to examine the global relevance of these findings. It would also be beneficial to use more precise dietary assessment tools, such as food diaries or biomarkers of sugar intake, to improve data accuracy. Research should further explore the impact of socioeconomic policies, such as sugar taxes and educational campaigns, on reducing sugar intake and improving oral health outcomes. Additionally, future studies should consider alternative explanations for dental caries, such as the role of other dietary factors (e.g., acidic beverages, processed foods), genetic predispositions, oral microbiota variations, and environmental influences (e.g., air pollution, water access). Furthermore, interdisciplinary approaches integrating oral health with broader nutritional and systemic health initiatives could amplify the effectiveness of public health strategies.

## 5. Conclusions

This study underscores the significant association between free sugar intake and dental caries in school-aged children in the U.S., with disparities driven by socioeconomic and demographic factors. The findings highlight the preventable nature of dental caries and the critical need for targeted public health interventions and policy measures to reduce sugar consumption and address health inequities. Policymakers, clinicians, and educators can leverage this evidence to implement strategies that promote healthier diets, improve access to dental care, and reduce the burden of dental caries in children. While addressing disparities and fostering healthier habits may contribute to improved oral health, further research is needed to confirm the long-term effectiveness of these strategies.

## Figures and Tables

**Table 1:** Mean DMFT score according to sociodemographic and health characteristics in NHANES cycles 2011–2016.

	*n* (%)		DMFT	
			Mean (±SE)	*p*-Value
Total	3658 (100)		1.69 (0.04)	NA
Sex				
Boys	1860 (50.8)		1.80 (0.06)	0.03 ^a^
Girls	1798 (49.2)		1.57 (0.06)	
Race and ethnicity				
Mexican American	769 (21)		2.16 (0.11)	<0.001 ^b^
Non-Hispanic Asian	296 (8.1)		1.78 (0.15)	
Non-Hispanic Black	942 (25.8)		1.73 (0.09)	
Non-Hispanic White	980 (26.8)		1.39 (0.08)	
Other Race ^1^	223 (6.4)		1.38 (0.16)	
Other Hispanic	438 (12)		1.55 (0.12)	
Family educational level				
<9th Grade	371 (10.4)		2.26 (0.15)	
9th–11th Grade	521 (14.7)		2.21 (0.13)	
High school graduate or GED	742 (20.9)		2.07 (0.1)	<0.001 ^b^
Some college	1084 (30.5)		1.50 (0.07)	
College graduate or higher	835 (23.5)		1.05 (0.07)	
Ratio of family income to poverty				
<1	1169 (34.0)		2.12 (0.08)	<0.001 ^a^
≥1	2274 (66.0)		1.47 (0.05)	
Last dental visit				
Within last year	3101 (84.8)		1.70 (0.05)	0.04 ^b^
More than 1 year but less than 5 years	436 (11.9)		1.77 (0.13)	
More than 5 years, never or does not know	121 (3.3)	1	1.14 (0.20)	
Could not get dental care (last year)				
Yes	200 (5.6)		2.6 (0.20)	<0.001 ^a^
No	3362 (94.4)		1.65 (0.05)	
Body Mass Index				
Underweight	112 (3.1)		1.88 (0.27)	0.76 ^b^
Normal weight	2182 (59.9)		1.73 (0.06)	
Overweight	586 (16.1)		1.69 (0.11)	
Obese	764 (21)		1.58 (0.09)	

^1^ Including multi-racial. ^a^ Mann–Whitney U test. ^b^ Kruskal–Wallis test.

**Table 2:** Association between dental caries experience and free sugar intake, NHANES, 2011–2016.

	Quantile Range	N	DMFT (±SE)	DMFT Rate Ratio (95% Confidence Interval)	
				Crude	Adjusted ^a^
Free Sugar Intake					
Quartile 1	0.00–37.60	916	1.50 (0.08) ^b^	Reference	Reference
Quartile 2	37.61–62.70	913	1.72 (0.09) ^b^	1.14 [1.02–1.28]	1.22 [1.08–1.38]
Quartile 3	62.71–97.00	914	1.60 (0.08) ^b^	1.07 [0.95–1.20]	1.18 [1.04–1.34]
Quartile 4	97.01–724.12	915	1.94 (0.09) ^b^	1.29 [1.15–1.45]	1.39 [1.23–1.58]

^a^ Adjusted for age, gender, race/ethnicity, poverty-to-income ratio, family educational level, last dental visit, ability to get dental care, and body mass index. ^b^ *p* < 0.05 Kruskal–Wallis test.

## Data Availability

The data for this study are publicly available at https://wwwn.cdc.gov/nchs/nhanes/Default.aspx.

## Supplementary Materials

The following supporting information can be downloaded at https://www.mdpi.com/article/10.3390/dj13020048/s1: Table S1: STROBE checklist for the study.
