# Supplementary material for: Impact of Free Sugar Consumption on Dental Caries: A Cross-Sectional Analysis of Children in the United States
Source: Dent J (Basel). 2025 Jan 22;13(2):48. doi: 10.3390/dj13020048 (PMC11854531; doi:10.3390/dj13020048)
Supplement: Supplementary file 1 [file dentistry-13-00048-s001.zip › dentistry-3388942-supplementary.pdf]

Supplementary Table S1: STROBE checklist for the study.

STROBE Checklist	Details
**Study Design**	Cross-sectional study utilizing data from NHANES cycles (2011–2012, 2013–2014, 2015–2016).
**Setting**	Data collected from mobile examination centers (MECs) across the U.S. during NHANES cycles. Recruitment occurred during home interviews and clinical evaluations.
**Participants**	Eligibility criteria: Children aged 6–12 with complete data on free sugar intake and oral health assessments. Participants were generally healthy, with mild to moderate conditions reflected in BMI and other health-related measures. No exclusions based on medical conditions.
**Variables**	Outcomes: DMFT (Decayed, Missing, and Filled Teeth) for dental caries. Exposures: Free sugar intake. Predictors: Age, gender, race/ethnicity, family educational level, last dental visit, ability to get dental care, BMI. Confounders: BMI controlled for in analysis.
**Data Sources/Measurement**	Data sources: NHANES 24-hour dietary recall interviews and dental exams. Free sugar intake quantified using FPED (Food Patterns Equivalent Database). DMFT measured using NHANES dental criteria. BMI from clinical measurements.
**Bias**	Efforts to address bias: Controlled for demographic and health-related variables, including BMI and access to oral health services.
**Study Size**	3,658 children (aged 6–12) with complete data on sugar intake and oral health from NHANES cycles 2011–2016. The sample size was based on data availability for the specific age range and variables of interest.
**Quantitative Variables**	Quantitative variables (e.g., DMFT, free sugar intake) analyzed by stratifying sugar intake into quartiles. DMFT scores were compared using non-parametric tests (Mann-Whitney U, Kruskal-Wallis).
**Statistical Methods**	Statistical methods: Poisson regression used to evaluate associations between sugar intake and DMFT, controlling for confounding variables (age, gender, race/ethnicity, family education, BMI, etc.).
